# A Case of White Swelling Cured by Electricity

**Published:** 1801-04

**Authors:** Joseph Lamb

**Affiliations:** Surgeon.


### *A Case of White Swelling cured by Electricity; by* JOSEPH LAMB, *Surgeon.*

A Middle aged woman being afflicted with a fcrophulous affection in the right knee, (commonly termed a white fwelling;) and being in low circumftances, was fent to the Northampton Infirmary,

Infirmary, where ſhe was under the care of Dr. Carr for almoſt two years. The uſual remedies were applied without effect: ſhe had alſo loſt her ſpeech, for which ſhe had ſeven cauſtics applied to her back, with bliſters, &c.—Theſe at firſt ſeemed to relieve her, but on taking the ſlighteſt cold, her ſpeech would go again and leave her quite hoarſe. After all the remedies had been tried without ſucceſs, and being of a very weak conſtitution, ſhe was thought to be an improper ſubject to undergo the operation of amputation, and was at length diſcharged, which took place a conſiderable time before I left London. She has been afflicted with this complaint about ſeven years altogether, and with great difficulty could ſhe walk with crutches. The latter end of laſt ſummer ſhe applied to me for advice, being in violent pain. It will readily be believed that I was at a loſs to know in what manner to proceed, ſhe having previouſly had ſuch excellent advice. The only thing that appeared to me at all likely to give her the leaſt relief was electricity, which I accordingly propoſed to her, and ſhe conſented to ſuffer a trial. I gave a few gentle ſhocks in a variety of directions through the affected part, and alſo thro' the cheſt, which to my great ſurpriſe reſtored her voice immediately, and it continued ſeveral weeks; but by taking a violent cold, ſhe loſt it again; however, two or three ſhocks recovered it. The pain and hardneſs in the knee began to decreaſe, which had been ſo long contracted. I repeated the electricity three times a week until the preſent time, and am happy to ſay that ſhe is quite recovered of her lameneſs, to the aſtoniſhment of all who knew her. The tumour is entirely diſperſed, and ſhe can walk without her crutches, either up ſtairs or down, which ſhe had not been able to do for ſeven years paſt.

*Kings Sutton, near Banbury, March* 14, 1801.

